# Isolated nausea and vomiting as the cardinal presenting symptoms of clozapine-induced myocarditis: a case report

**DOI:** 10.1186/s12888-020-02955-9

**Published:** 2020-11-27

**Authors:** M. Z. van der Horst, F. van Houwelingen, J. J. Luykx

**Affiliations:** 1 Department of Psychiatry, UMC Utrecht Brain Center, University Medical Center Utrecht, Utrecht University, Utrecht, The Netherlands; 2 Department of Translational Neuroscience, UMC Utrecht Brain Center, University Medical Center Utrecht, Utrecht University, Utrecht, The Netherlands; 3 Outpatient Second Opinion Clinic, GGNet Mental Health, Warnsveld, The Netherlands

**Keywords:** Clozapine, Myocarditis, Clozapine-induced myocarditis, Schizophrenia, Case report

## Abstract

**Background:**

Clozapine is an atypical antipsychotic proven to be superior in the treatment of treatment-resistant schizophrenia. Myocarditis is a rare, but well-known complication of treatment with clozapine. Only few cases have been reported in which nausea and vomiting were prominent symptoms. This is the first described report in which nausea and vomiting were the only presenting symptoms of clozapine-induced myocarditis.

**Case presentation:**

We report a case of a 58-year-old woman, suffering from schizoaffective disorder, who is being treated with clozapine. Two weeks after initiation of clozapine, she developed nausea and vomiting, in absence of any other clinical symptoms. Laboratory examination and magnetic resonance imaging confirmed the diagnosis of clozapine-induced myocarditis. Clozapine was discontinued and the patient recovered fully.

**Conclusions:**

This case emphasizes the importance of recognizing myocarditis as a cause of isolated nausea and vomiting in patients treated with clozapine. Early recognition improves clinical outcome and reduces mortality.

## Background

Clozapine is the most effective treatment in patients suffering from psychotic disorders. Furthermore, it is the only antipsychotic agent that reduces death from suicide [1]. However, clozapine is the most notorious antipsychotic agent associated with the development of myocarditis. Clozapine-induced myocarditis is a rare but life-threatening condition; mortality rates range from 10 to 30% [2–5].

Myocarditis is defined as an inflammation of the heart muscle (myocardium) and has various causes [6]. The most common cause of myocarditis is viral infection, particularly parvovirus B19 and herpes simplex virus [7]. Other types of infections, auto-immune diseases, and hypersensitivity or toxic reactions to drugs can cause this condition as well [6]. Due to underdiagnoses and a lack of shared standardized diagnostic approach worldwide, the true incidence of myocarditis remains unclear [8]. In patients using clozapine the reported incidence worldwide varies from < 0.1 to 3.2% [4, 5, 9]. In Australia incidence rates up to 8.5% are reported, possibly due to genetic factors, geographical differences and/or greater awareness and monitoring [10–12]. Bellissima et al. (2018) found in their systematic review that in 87% of cases, symptoms of myocarditis displayed in the first 30 days of treatment [13]. Almost half of these patients developed symptoms in the first 12 days of treatment. However, some cases have been described in which patients developed clozapine-induced myocarditis 2 years after starting this therapy [3]. The risk of developing clozapine-induced myocarditis does not appear to be dose-dependent [13].

The pathophysiological mechanism of clozapine-induced myocarditis has not been elucidated. In 1999 Kilian et al. suggested that clozapine-induced myocarditis results from an IgE-mediated hypersensitivity reaction, since the development of eosinophil infiltrates and damaged myocytes corresponds to the time path of a type 1 allergic reaction and duration of the clozapine treatment [4]. Such eosinophilic drug reactions in the myocardium have also been described by methyldopa, hydrochlorothiazide, azithromycin, furosemide, benzodiazepines and tricyclic antidepressants [14–16]. Histologically, a pattern of interstitial infiltration with prominent eosinophilitis can be found [17]. Wang et al. (2008) studied clozapine-induced myocarditis in mice and found increased levels of pro-inflammatory cytokines, as was hypothesized before by previous research [18–20]. Furthermore, a significant dose-related increase in myocardial inflammation correlated with plasma catecholamine levels was found, as well as release of TNF-alpha. These findings might provide future treatment options, such as beta-adrenergic blocking agents.

It is known that the clinical presentation of myocarditis can vary widely. Most reported symptoms are shortness of breath (42%), chest pain (37%), flu-like symptoms (18%), cough (12%), and gastrointestinal discomfort (11%) [13]. Here, we report a case in which nausea and vomiting were the only signs leading to the diagnosis of clozapine-induced myocarditis. A systematic literature search provided no previous published reports where these symptoms were the only cardinal presenting signs. For this reason, this is the first case report describing nausea and vomiting as the only presenting symptoms of clozapine-induced myocarditis.

## Case presentation

A 58-year-old woman with a history of schizoaffective disorder, was admitted to our hospital for treatment of a psychotic episode with depressive symptoms, characterized by anxiety, nihilistic and paranoid delusions, depressive mood, bradyphrenia, and inability to complete activities of daily living. She did not experience hallucinations or suicidal thoughts. The patient’s medical history revealed no relevant diseases other than renal dysfunction after lithium therapy. Besides a tachycardia of 132 bpm (most likely due to anxiety), no other somatic abnormalities were found in physical examination on admission. The patient was using lamotrigine (200 mg/day), olanzapine (20 mg/day), and quetiapine (300 mg/day). Notable past medication included lithium, which was stopped 3 years earlier because of renal insufficiency. Since the current pharmacological treatment was insufficiently effective, olanzapine was gradually switched to clozapine. Lamotrigine and quetiapine were continued for its antidepressive effect. Clozapine was initiated on day 1 in a dosage of 12.5 mg twice a day and titrated according to prescribing recommendations. On day 15 she reached a dosage of 300 mg/day.

On day 18 she complained about nausea and vomited once. The pre-existing tachycardia was still present, with a heart rate of 111 bpm. Further physical examination was without abnormalities. She did not suffer from diarrhea, abdominal cramps or pains, loss of appetite, or fever. Differential diagnosis at this point included gastrointestinal infection, constipation, drug-induced nausea, myocardial infarction, and (clozapine-induced) myocarditis. Although gastrointestinal infection is the most likely cause of nausea and vomiting and suspicion for myocardial infarction and (clozapine-induced) myocarditis at this point was low, further diagnostic investigation was initiated to rule out these potentially life-threatening conditions. Non drug-related causes of myocarditis, such as viral infections, were less likely because of the lack of symptoms associated with viral disease and the arise of symptoms shortly after the initiation of clozapine. Besides clozapine, few cases have been described in which lamotrigine or quetiapine was the cause of drug-induced myocarditis [21–24]. However, our patient was using lamotrigine and quetiapine long before the symptoms occurred (> 1 year), making it far less likely to be the current cause of the myocarditis.

Laboratory results showed leukocytosis, eosinophilia, elevated troponin I, creatine kinase, and monocytes (Table 1). Electrocardiogram (ECG) showed a sinus rhythm of 103 bpm, normal heart axis, slight PTa depression inferior in V5-V6, and no signs of ischemia.

**Table 1:** Laboratory results

Laboratory measurement	Result	Normal range	Units of measurement
Hemoglobin	8.2	7.4–9.6	mmol/l
Leukocytes	**11.0**	4.0–10.0	× 10^9^/l
Thrombocytes	244	150–450	×10^9^/l
Creatinine	**161**	49–90	μmol/l
Troponin I	**122**	0–45	ng/l
Creatine Kinase	**315**	0–145	U/l
Eosinophilic granulocytes	**0.46**	0.00–0.40	×10^9^/l
Basophilic granulocytes	0.01	0.00–0.20	× 10^9^/l
Neutrophilic granulocytes	8.16	1.60–8.30	×10^9^/l
Lymphocytes	1.50	0.80–4.00	×10^9^/l
Monocytes	**0.86**	0.20–0.80	×10^9^/l

An ultrasound of the heart showed a non-dilated left ventricle with normal systolic function, but abnormal relaxation. The right ventricle had a normal dimension and function. There was a suspicion of minimal compression of the right ventricular outflow tract and little pericardial effusion of the right ventricle was seen.

Considering symptomatology, lab results (elevated heart enzymes and eosinophilia), and radiology findings (pericardial effusion), there was a high clinical suspicion of clozapine-induced myocarditis and clozapine was discontinued abruptly. Cardiac magnetic resonance imaging (MRI) confirmed the diagnosis. It showed edema in the myocardium, which is a sign of (early) myocarditis. There was no delayed enhancement or signs of pericarditis. Little pericardial effusion and a normal systolic function of both ventricles was seen.

After discontinuation of clozapine, the nausea improved. Troponin levels returned to normal and the heart ultrasound showed no abnormalities, except for minimal pericardial effusion of the right ventricle. No further cardiological follow-up was required. The patient was switched to amisulpride, but this did not improve her symptoms. Ultimately, symptoms diminished after electroconvulsive therapy.

## Discussion and conclusions

Our patient experienced nausea and vomiting in absence of chest pain or shortness of breath. A systematic search was performed in Pubmed (last updated on 12 June 2020) using the following terms: clozapine, leponex, clozaril, clozapine-induced myocarditis, myocarditis, nausea, and vomiting. The search yielded one result; a case report of a patient who suffered from nausea, and in addition, fever, dyspnea, fatigue, sinus tachycardia, elevated troponin, and C-reactive protein (CRP) levels, and reduced ejection fraction, leading to the diagnosis of clozapine-induced myocarditis [25]. When studying (systematic) reviews, a few cases were found in which nausea and vomiting were prominent symptoms [26–28]. However, of these cases, only two presented without any cardiopulmonary symptoms. These cases have not been yielded by our systematic search, because in one, the case is only mentioned as part of the review and in the other, the case is not described in detail but is part of a summary of other cases of clozapine-induced myocarditis. In the case report of Caetano and Ghandi (2008), ‘feeling feverish’ was reported as the only extra presenting symptom [26]. Hägg et al. (2001) described a patient suffering from nausea and vomiting in addition to influenza-like disease, fever, exanthema, tachycardia, confusion, and stroke [28]. Another patient from the same study experienced nausea and vomiting as well, but no other symptoms were described. An unexpected sudden death is reported in this patient. Autopsy confirmed the diagnosis of myocarditis [28]. However, the case is not described in detail. This makes our case report unique, since it is the only case of a patient with nausea and vomiting as the only presenting symptoms of clozapine-induced myocarditis described in detail. Several cases have been described in which patients remained asymptomatic and in some cases only post-mortem analysis led to the diagnosis of myocarditis [5, 13, 29].

When clozapine-induced myocarditis is suspected, further examination is essential for early detection and intervention. In more than half of described cases, one or more of the following clinical findings were reported: fever, tachycardia, elevated CRP levels, eosinophilia, elevated cardiac markers, and elevated troponin levels [13]. Less commonly reported are hypotension, tachypnea, lung crepitation, S3 and/or S4 heart sound, and ECG changes (most reported are ST-segment elevation and T-wave inversions). As these findings are often nonspecific, additional research by echocardiogram and especially cardiac MRI is recommended. Most reported findings include ventricular dilatation and/or dysfunction, pericardial effusion, edema, and late gadolinium enhancement of the myocardium [13, 30].

After diagnosis of clozapine-induced myocarditis the first step is to discontinue the drug immediately and to consult a cardiologist [31]. Treatment with diuretics, angiotensin converting enzyme inhibitors, beta-blockers and steroids might be needed [13]. In general, future rechallenge of clozapine is not recommended due to the high risk of recurrence of cardiac problems [32, 33]. However, due to the lack of an equally effective alternative antipsychotic, sometimes rechallenge is embarked, after carefully weighing the benefits and risks. Shivakumar et al. (2019) proposed a protocol for clozapine rechallenge in which they suggest a strategy of slow titration combined with interruption of titration once any indices increase (monocytes, neutrophils, eosinophils, or CRP) [32].

Further research to identify the risk factors associated with clozapine-induced myocarditis may enable personalized medicine to better estimate which patients will experience serious side effects, and may reduce the delay in starting this most effective drug therapy [34]. Ronaldson et al. (2012) identified clinical and phenotypic risk factors for clozapine-induced myocarditis and found the risk to be increased in the case of rapid dose titration, (odds ratio (OR) 1.26, 95% confidence interval (CI) 1.02–1.55, *p* = 0.03), concomitant use of sodium valproate (OR 2.59, CI 1.51–4.42, *p* = 0.001), and ascending age (OR 1.31, CI 1.07–1.60, *p* = 0.009) [35]. These risk factors accounted for less than 50% of the risk of clozapine-induced myocarditis, and it was hypothesized that genetic factors might be responsible for another substantial portion of the risk. The same research group performed a genome-wide association study and human leucocyte antigen (HLA) analyses and identified four single nucleotide proteins to be associated with increased risk of myocarditis and two HLA alleles [36]. Polygenic risk score based on variation at 96 different genetic sites explained 66% of liability (*p* = 9.7 × 10^-5) [33]. The combination of both clinical and genetic factors provided the highest increase in proportion of variability accounted for (r^2^ 0.73, *p* = 9.8 × 10^-9) [34]. Further research with larger sample sizes is needed for validation.

Summarizing, when treating patients with clozapine, it is crucial to be aware of the risks of this drug and to know how to manage them. Clinical presentation of clozapine-induced myocarditis is often nonspecific and nausea and vomiting can be the only symptoms present (as it was in our patient). Early recognition and intervention can prevent further myocardial damage and safe lives. However, the risk of myocarditis should not create a barrier to start with clozapine in patients who can benefit from this highly effective drug treatment. In the future, identification of phenotypic and genotypic risk factors may enable personalized medicine to reduce the risk of developing serious adverse events.

In conclusion, we recommend to seriously consider clozapine-induced myocarditis as a cause of nausea and vomiting in every patient in the first month after initiation of clozapine, because of the relatively high occurrence of myocarditis in the first 30 days of treatment. This recommendation applies regardless of the patient’s age, gender, or history. Further diagnostic work-up with laboratory testing and electrocardiography should always be performed.

## Data Availability

Not applicable.

## Abbreviations

ECG: Electrocardiogram
MRI: Magnetic Resonance Imaging
CRP: C-reactive Protein
OR: Odds Ratio
CI: Confidence Interval
HLA: Human Leucocyte Antigen
